# How Does a Simple Network of Chemical Oscillators See the Japanese Flag?

**DOI:** 10.3389/fchem.2020.580703

**Published:** 2020-11-09

**Authors:** Jerzy Gorecki, Ashmita Bose

**Affiliations:** Department of Complex Systems and Chemical Processing of Information, Institute of Physical Chemistry, Polish Academy of Sciences, Warsaw, Poland

**Keywords:** chemical oscillating reaction, Belousov-Zhabotinsky, Oregonator model, mutual information, evolutionary optimization, network, chemical computation

## Abstract

Chemical computing is something we use every day (e.g., in the brain), but we can still not explore and master its potential in human-made experiments. It is expected that the maximum computational efficiency of a chemical medium can be achieved if information is processed in parallel by different parts of the medium. In this paper, we use computer simulations to explore the efficiency of chemical computing performed by a small network of three coupled chemical oscillators. We optimize the network to recognize the white and red regions of the Japanese flag. The input information is introduced as the inhibition times of individual oscillators, and the output information is coded in the number of activator maxima observed on a selected oscillator. We have used the Oregonator model to simulate the network time evolution and the evolutionary optimization to find the best network for the considered task. We have found that even a network of three interacting oscillators can recognize the color of a randomly selected point with 95% accuracy.

## 1. Introduction

The success of semiconductor technology in machine information processing is the consequence of a highly efficient realization of logic gates characterized by a long time of error-free operation. The gates can be downsized to the nanoscale and concatenated to make more complex information processing devices. The semiconductor technology perfectly matches the bottom-up design scenario of information processing systems according to which more complex operations are represented by the combination of simpler tasks for which constructions of corresponding circuits have already been developed (Feynman et al., 2000).

The usefulness of logic gates and binary information coding demonstrated by semiconductor devices has strongly influenced other fields of unconventional computation including the chemical one (Adamatzky, 2018). Many studies have focused on strategies for the use of binary information coding in a computing medium and on realization of the logic gates or binary operations (Hjelmfelt et al., 1992; Adamatzky and De Lacy Costello, 2002; Magri et al., 2006; de Silva and Uchiyama, 2007; Gorecki et al., 2009). In some cases, molecular logic gates used as molecular probes offer an interesting alternative to the standard techniques (McKinney et al., 2017). However, most proposed chemical gates were much less efficient and were slower than the equivalent operations performed using semiconductors. The fact that a medium allows us to generate all logic gate proofs that the universal computation with this medium is theoretically possible. However, usually, the gates made of a chemical computing medium have limited potential applications. In the case of the Belousov-Zhabotinsky (BZ) reaction (Belousov, 1959; Zhabotinsky, 1964), the output signal appears a few seconds after the input is introduced (Toth and Showalter, 1995; Steinbock et al., 1996). For other media, this time can be longer. For example, for gates with information coded in DNA molecules, it may take a few hours before the gate answer is obtained (Lin et al., 2018). The bottom-up construction of chemical information processing devices does not therefore seem to lead to an efficient realization of algorithms.

On the other hand, living organisms use chemistry for information processing and do so with significant efficiency for various classes of algorithms, including sound and image recognition, orientation in space, or navigation in crowded environments. This observation demonstrates that a chemical medium can be efficiently applied for specific computing tasks and presumably solve them using a highly parallel approach. Applications of parallel chemical computation have been reported in the literature. The classic example is the Adleman demonstration that the Hamiltonian path problem can be solved with DNA molecules (Adleman, 1994; Calude, 2002). Another example is the so-called prairie-fire algorithm for verification if there is a path linking two randomly selected points in a labyrinth. This problem can be solved by a labyrinth formed of an excitable medium where stable pulses of excitation can propagate (Steinbock et al., 1995; Agladze et al., 1997). If there is a path linking two points, an excitation generated at one of the points will then appear at the other, and the time difference between excitation and detection gives the estimation for the shortest path linking these points. Yet another famous computing application of a chemical medium working in parallel is the image processing of black and white photos performed using a photosensitive variant of BZ-reaction proceeding in a uniform, spatially distributed system (Kuhnert, 1986, 1989; Rambidi and Maximychev, 1997). In such medium, image processing is the consequence of a non-homogeneous initial state generated by initial illumination with intensity proportional to the grayscale of pixels of the processed image. In all methods mentioned above, the output information is coded in the time evolution of the computing medium.

The number of examples where a chemical medium can be efficiently used for computing is, however, limited. A top-down design strategy offers a promising method for the identification of the new ones. The strategy can be summarized as follows. In the beginning, we select a problem we want to solve and the computing medium that is supposed to do it. Next, we define how the input information is introduced and how the output is extracted from the observation of medium evolution. The top-down approach can be applied if the properties of the medium—and thus of the medium evolution—can be controlled by a number of adjustable parameters. Within this strategy, we are supposed to find the values of parameters for which the medium answer (the output) gives the most accurate solution of the considered problem. To do this, we need a number of examples (the training dataset) that can be used to verify the accuracy of computation performed by the medium.

**Figure 1:**
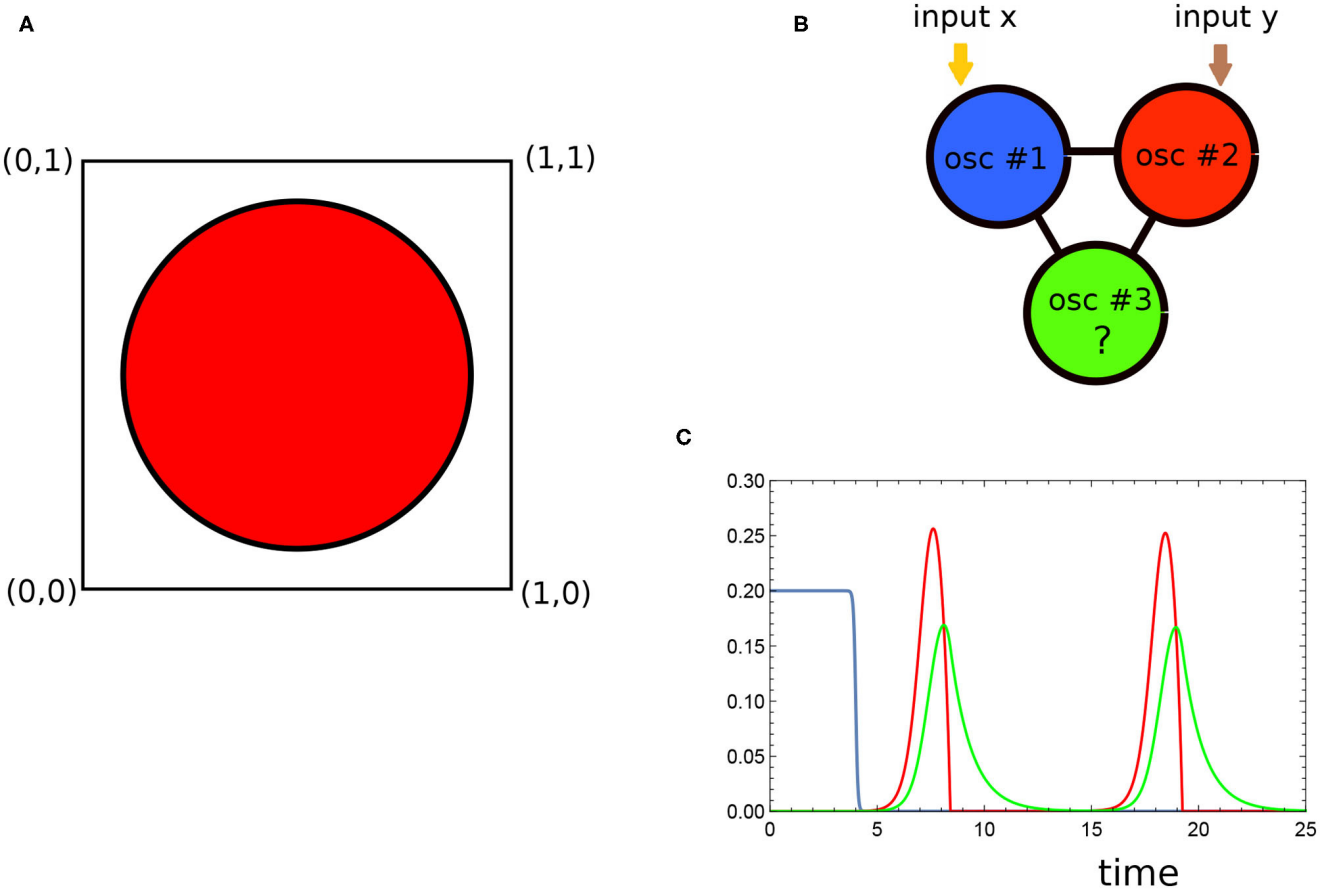
**(A)** The graphical illustration of the problem that is solved by the optimized network. The disk is located at the square center. Its radius was selected such that the areas of the red sun and the white surrounding are equal. The network is supposed to answer if a randomly selected point (*x, y*) ∈ [0, 1] × [0, 1] belongs to the red or to the white regions. **(B)** The structure of the considered chemical oscillator network. The black line indicates interactions between oscillators. The type of oscillator #3 was optimized to achieve the maximum mutual information between locations of points and the number of activator maxima. **(C)** The time evolution of the activator (the red curve) and the inhibitor (the green line) for the Oregonator model with the activator decay corresponding to that of the optimized network (α = 0.849). The blue line represents the external illumination (cf. Equation 4, *t*_*illum*_ = 4).

In this paper, we concentrate on the geometrically oriented problem illustrated in Figure 1A. The object of our research is the Japanese flag with slightly rescaled proportions. We consider a red disk (sun) located in the mid of a white square (here represented by the Cartesian product [0, 1] × [0, 1]). We postulate that a chemical computer can answer if a randomly selected point (*x, y*) ∈ [0, 1] × [0, 1] is located in the red or in the white region. To make the problem difficult, the disk radius $r={\left(\sqrt{2\pi }\right)}^{-1}$ is selected such that the areas of the sun and the white region are equal. A device that gives a random answer or a device that gives the same answer (red, white) to all inputs thus solves the problem with 50% accuracy (or with 50% chance to obtain the wrong answer). We show below that a chemical medium can solve the problem with much higher accuracy.

We postulated that the chemical medium composed of three coupled oscillators, illustrated in Figure 1B, was able to produce an accurate solution to the problem. The two-variable Oregonator model (Field and Noyes, 1974; Jung et al., 1998) of the Belousov-Zhabotinsky reaction (cf. Equations 1, 2) was used to describe the time evolution of individual oscillators. The interactions between individual oscillators were represented by reactions involving activators of individual oscillators. The choice of model has been motivated by the broad interest in applications of BZ-reaction for chemical information processing. The BZ-reaction is a complex, catalytic oxidation of an organic substrate (usually malonic acid) in an acidic environment (Field and Burger, 1985; Epstein and Pojman, 1998). Two stages of BZ reaction can be identified. One is fast oxidation of the catalyst, and the other is a slow reduction of the catalyst by an organic substrate. The solution color reflects concentrations of catalyst in the oxidized and reduced forms, and such types of non-linear evolution of the medium as oscillations, wave propagation, or the appearance of spatio-temporal patterns can therefore be easily observed. If the ruthenium complex ($Ru{\left(bpy\right)}_{3}^{2+}$) is used as the reaction catalyst, the BZ-reaction then becomes photosensitive (Kádár et al., 1997) and can be externally controlled by illumination. For the same initial concentrations of reagents, the medium can oscillate at dark, show an excitable behavior at a low light intensity, and have a steady state when it is strongly illuminated. At specific conditions, a spatially distributed medium can be locally excited, and the excitation can propagate in space. This type of behavior resembles the propagation of nerve impulses in living organisms. As a result, the BZ reaction has attracted attention as an inexpensive medium for experiments with neuron-like chemical computing (Adamatzky et al., 2005; Gorecka and Gorecki, 2006; Gentili et al., 2017). A moving pulse of excitation can be interpreted as a propagating bit of information. The Oregonator model used below to simulate *in-silico* the time evolution of the medium (see Equations 1, 2) correctly describes this phenomenon (Holley et al., 2011). However, such an approach requires a spatially distributed medium with a complex structure and precisely controlled reaction parameters, which seems complicated in real applications (Adamatzky et al., 2011).

In a number of recent papers, we considered flow of information (Grüunert et al., 2015) and the computational potential of coupled oscillator networks (Gizynski and Gorecki, 2016, 2017b; Gizynski et al., 2017). It was demonstrated that network parameters could be adjusted such that it can solve selected problems, e.g., recognizing a sphere in a multidimensional space or concluding on the type of cancer on the basis of results of medical tests, with high accuracy. However, numerical simulations leading to these conclusions were based on oversimplified event-based-model reflecting only the basic features of the time evolution of a chemical oscillator and interactions between oscillators coupled with mutual activations. The event-based-model assumes sharp boundaries between three phases of the oscillation cycle: excitation, refractory, and responsive phases. It takes interactions into account as the condition for the excitation of an oscillator in the responsive phase in contact with an excited oscillator. Here, we present simulation results using the Oregonator model for a single oscillator and a model for oscillator interactions based on reactions involving their activators. We therefore believe the current model is more realistic than the one previously used and gives more information for potential experiments on the chemical computation.

The paper is organized as follows. The description of the mathematical model of the network time evolution and the optimization procedure are described in the next section. Section 3 contains obtained results and their discussion. The conclusions summarize obtained results and present suggestions for the future studies.

## 2. The Model of the Oscillator Network and Its Optimization Procedure

### 2.1. The Network

We postulate that the problem of attributing color to a point on the Japanese flag defined by its coordinates can be approximately solved by a network of interacting chemical oscillators (cf. Figure 1B). The time evolution of each individual oscillator is described by two-variable Oregonator model (Field and Noyes, 1974; Jung et al., 1998) combined with additional reaction (cf. Equation 3) that reduces the concentration of activator. Let us assume that the variables *u*_*j*_ and *v*_*j*_ represent concentrations of the activator (*U*_*j*_) and the inhibitor (*V*_*j*_) for reactions proceeding in the oscillator #j. The equations describing the time evolution of *u*_*j*_ and *v*_*j*_ read:

(1) $$\begin{array}{l} \frac{\partial u_{j}}{\partial t}=\frac{1}{\varepsilon }\left(u_{j}-u_{j}^{2}-\left(fv_{j}+\phi_{j}\left(t\right)\right)\frac{u_{j}-q}{u_{j}+q}\right)-\alpha u_{j} \end{array}$$

(2) $$\begin{array}{l} \frac{\partial v_{j}}{\partial t}=u_{j}-v_{j} \end{array}$$

The parameter ε sets up a ratio of time scales of variables *u*_*j*_ and *v*_*j*_, and *q* is a scaling constant and *f* is the stoichiometric coefficient. In our simulations we used the following values of Oregonator model parameters for all oscillators (1 ≤ *j* ≤ 3): ε = 0.2, *q* = 0.0002 and *f* = 1.1. These values are similar to those reported in the literature (Holley et al., 2011). We assumed that the parameters of the Oregonator model were fixed and did not undergo optimization when we searched for the network with the highest ability to relate point position with its color.

The last term in Equation (1) describes the additional decay of activator with the reaction rate α, which can be represented by the following reaction:

(3) $$\begin{array}{l} U_{j}+D_{j}\to products \end{array}$$

The time dependent function ϕ_*j*_(*t*) describes the influence of illumination on a photosensitive BZ-reaction and it is proportional to the light intensity. We considered ϕ_*j*_(*t*) in the following form:

(4) $$\begin{array}{l} \phi_{j}\left(t\right)=0.1\cdot (1.001+\tanh\left(-10\left(t-t_{ilum}\left(j\right)\right)\right) \end{array}$$

In this definition, the parameter *t*_*ilum*_(*j*)(> 0) defines the moment at which the illumination of the *jth* oscillator is switched off. At the beginning, the value of ϕ_*j*_(*t*) ~ 0.2 and the Oregonator model, with parameters given above and without the additional decay of the activator, predicts a stable steady state corresponding to *u*_*j*_ = 0.0002 and *v*_*j*_ = 0.0002. For long durations, ϕ_*j*_(*t*) approaches 0.0001, which corresponds to an oscillation with a period of approximately 10.8 time units. The time evolution of the activator (the red curve) and inhibitor (the green line) for the Oregonator model for α = 0.849 is illustrated in Figure 1C. The blue line represents the external illumination for *t*_*illum*_ = 4.

We assumed that oscillators in the network are coupled via the transport of the activator. This type of coupling was observed in our experiment on BZ-droplets stabilized by a solution of lipids in decane (Szymanski et al., 2011; Gizynski and Gorecki, 2017a). However, the network optimization method discussed below is general and can be applied to other couplings, such as the inhibitory coupling via transport of bromine studied by Vanag and his co-workers (Vanag and Epstein, 2004; Kaminaga et al., 2005, 2006; Smelov and Vanag, 2017). In such a case, a more complex model of oscillator dynamics should be applied (Vanag and Yasuk, 2018).

The interactions between oscillations in the considered network are indicated by the linking lines shown in Figure 1B. The pairs oscillators that interacted were fixed, and we did not modify the interactions during the network optimization. The coupling between the oscillators #k and #j was described by additional reactions involving the activators *U*_*k*_ and *U*_*j*_ of these oscillators:

(5) $$\begin{array}{l} U_{j}+B_{j}\to U_{k}+C_{k} \end{array}$$

(6) $$\begin{array}{l} U_{k}+B_{k}\to U_{j}+C_{j} \end{array}$$

with the identical reaction rate β. In reactions (3,5,6) *B, C*, and *D* denote other molecules involved. We assume their concentrations were high with respect to concentrations of activators involved. The concentrations of *B* and *D* were thus assumed to be constant during the network evolution, and they are consequently not included in the model. Such an approach reflects the idea that reactions in individual oscillators are independent, and their coupling occurs as the result of processes 5 and 6 only.

Within our model the time evolution of the network is described by the following set of kinetic equations:

(7) $$\begin{array}{l} \frac{\partial u_{j}}{\partial t}=\frac{1}{\varepsilon }\left(u_{j}-u_{j}^{2}-\left(fv_{j}+\phi_{j}\left(t\right)\right)\frac{u_{j}-q}{u_{j}+q}\right)-\left(\alpha +3\beta \right)u_{j}+\beta \left(u_{1}+u_{2}+u_{3}\right) \end{array}$$

(8) $$\begin{array}{l} \frac{\partial v_{j}}{\partial t}=u_{j}-v_{j} \end{array}$$

Let us notice that for each index *j* the contributions β*u*_*j*_ in the last two terms in Equation (7) cancel out reduce to $-\left(\alpha +2\beta \right)u_{j}+\underset{i\neq j}{\sum }\beta u_{i}$, which reflects the kinetics of processes (3), (5), and (6).

Mathematically, the terms resulting from processes (5) and (6) have a similar form to those describing the coupling between CSTRs resulting from the exchange of equal volumes of reagents (and assuming that the is no transport of inhibitor). For the considered parameters of the Oregonator model (as well as a few other we tried), we found the interactions between oscillators described by reactions (5,6) were difficult to control without the process (3) because the model, depending on the value of β, gave too weak or too strong coupling between oscillators. The introduction of reaction (3) allowed us to control the value of activator concentration around its maximum and to moderate these interactions without the need to optimize all parameters of the Oregonator model.

Following our previous studies, we assume that an oscillator in the network can perform one of two functions (Gizynski and Gorecki, 2016, 2017b; Gizynski et al., 2017). There are input oscillators used to introduce the input values into the network. The activity of an oscillator assigned as the input of *x* or *y* is suppressed for time related to the input value. We assume that the relationship is described by an affine function. If the *jth* oscillator functions as the input one for the coordinate *x* ∈ [0, 1], then

(9) $$\begin{array}{l} t_{ilum}\left(j\right)=t_{start}+\left(t_{end}-t_{start}\right)\cdot x \end{array}$$

Keep in mind the symmetry of the problem the input oscillator #k for the *y* value is inhibited for time:

(10) $$\begin{array}{l} t_{ilum}\left(k\right)=t_{start}+\left(t_{end}-t_{start}\right)\cdot y, \end{array}$$

and the values of parameters *t*_*end*_ and *t*_*start*_ in Equations (9) and (10) are identical. The values of *t*_*end*_ and *t*_*start*_ were the subject of network optimization procedure. The relationship between *t*_*ilum*_ and the input value, obtained for the Japanese flag problem, is illustrated in **Figure 3B**.

The network can also include so-called normal oscillators inhibited for a fixed time that is not related to the values of *x* and *y*. These normal oscillators moderate interactions in the medium and optimize it for a specific problem. Here, the type of oscillator #3 was not fixed, and the optimization procedure decided it.

We also assumed that the output information is coded in the number of activator maxima observed on one of the network oscillators within the time interval [0, *t*_*max*_]. As we show later, the choice of the output oscillator results directly from the network optimization. The full definition of a computing network therefore includes the number of oscillators in the network, their types, the method of inputting *x*, *y* values, and the interactions between oscillators.

The parameters that were modified during the network optimization procedure were:

- The type of oscillator #3 and, in the case it was a normal oscillator, its illumination time *t*_*ilum*_(3),
- The length of time interval *t*_*max*_, within which the network evolution was observed,
- The times *t*_*start*_ and *t*_*end*_,
- The reaction rates α and β.

**Figure 2:**
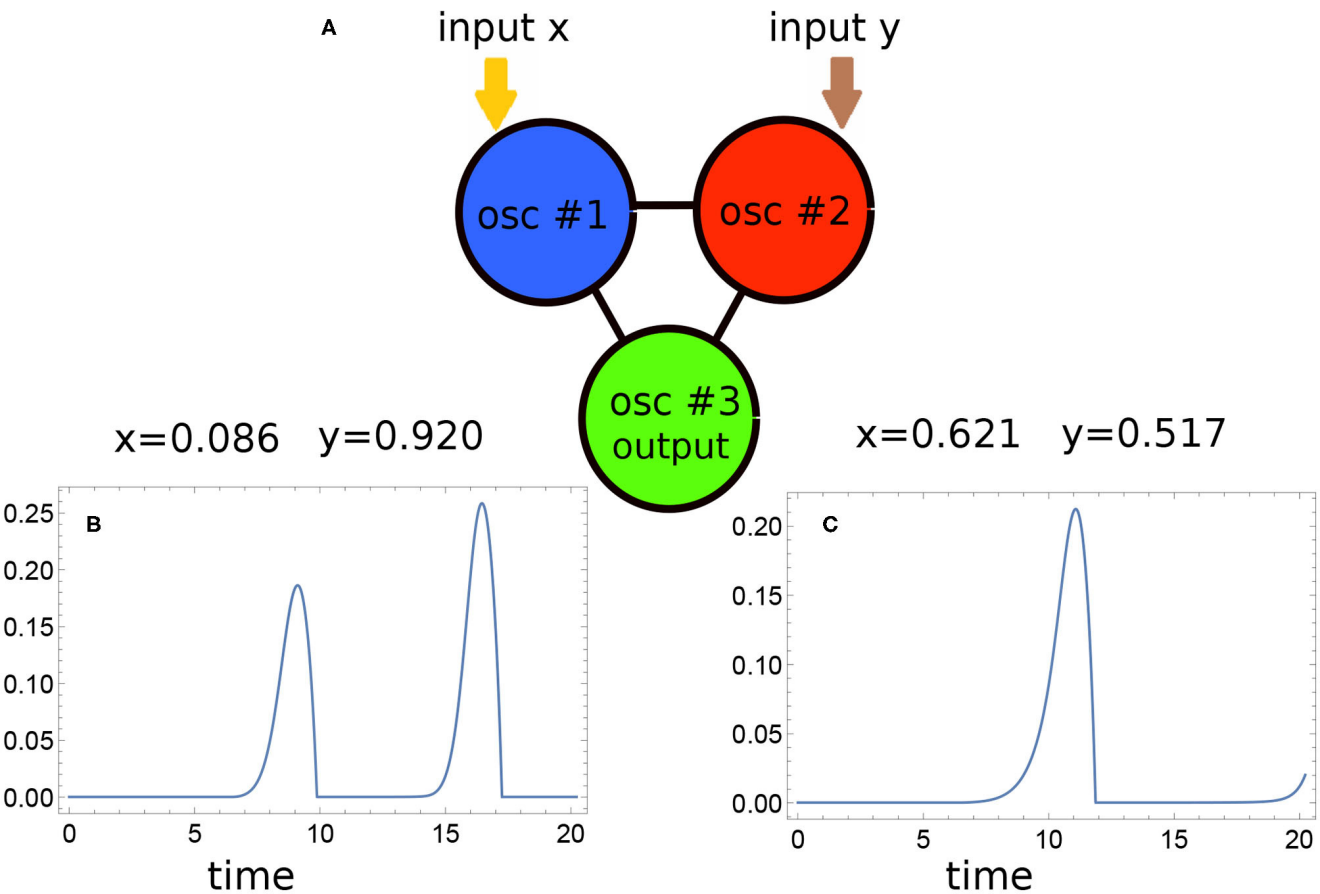
**(A)** The idea of information processing with a network of oscillators illustrated using the parameters of the optimized network. Oscillators #1 and #2 are inhibited by times related to the values of *x* and *y* respectively. The output information is coded in the number of activator maxima observed on the oscillator #3. The time evolution of activator observed on this oscillator for points located in the white and red regions are shown in **(B,C)**, respectively. The evolution was calculated using the parameters of the optimized network: the oscillator #3 is the normal one with *t*_*ilum*_(3) = 6.37, *t*_*max*_ = 20.23, *t*_*start*_ = 3.78, *t*_*end*_ = 12.10, α = 0.849, β = 0.251.

The values of network parameters and the parameters describing the relationship between the input values and the time-dependent illuminations of input oscillators determine the medium evolution. For each set of parameters, we solved the set of Equations (7, 8) numerically and studied the time evolution of the network for any values of *x* and *y*. We used the explicit fourth-order Runge-Kutta algorithm (Press et al., 2007) with *h* = 10^−4^ time step. The output information corresponding to a specific input was extracted from the numerical solution as the number of activator maxima observed at the selected oscillator within the time interval [0, *t*_*max*_]. Figures 2B,C show such evolution for two selected points, one outside the sun and the other inside it. In the first case, the output oscillator produced two maxima of activator. In the second, we observed just a single one.

### 2.2. Network Optimization

**Figure 3:**
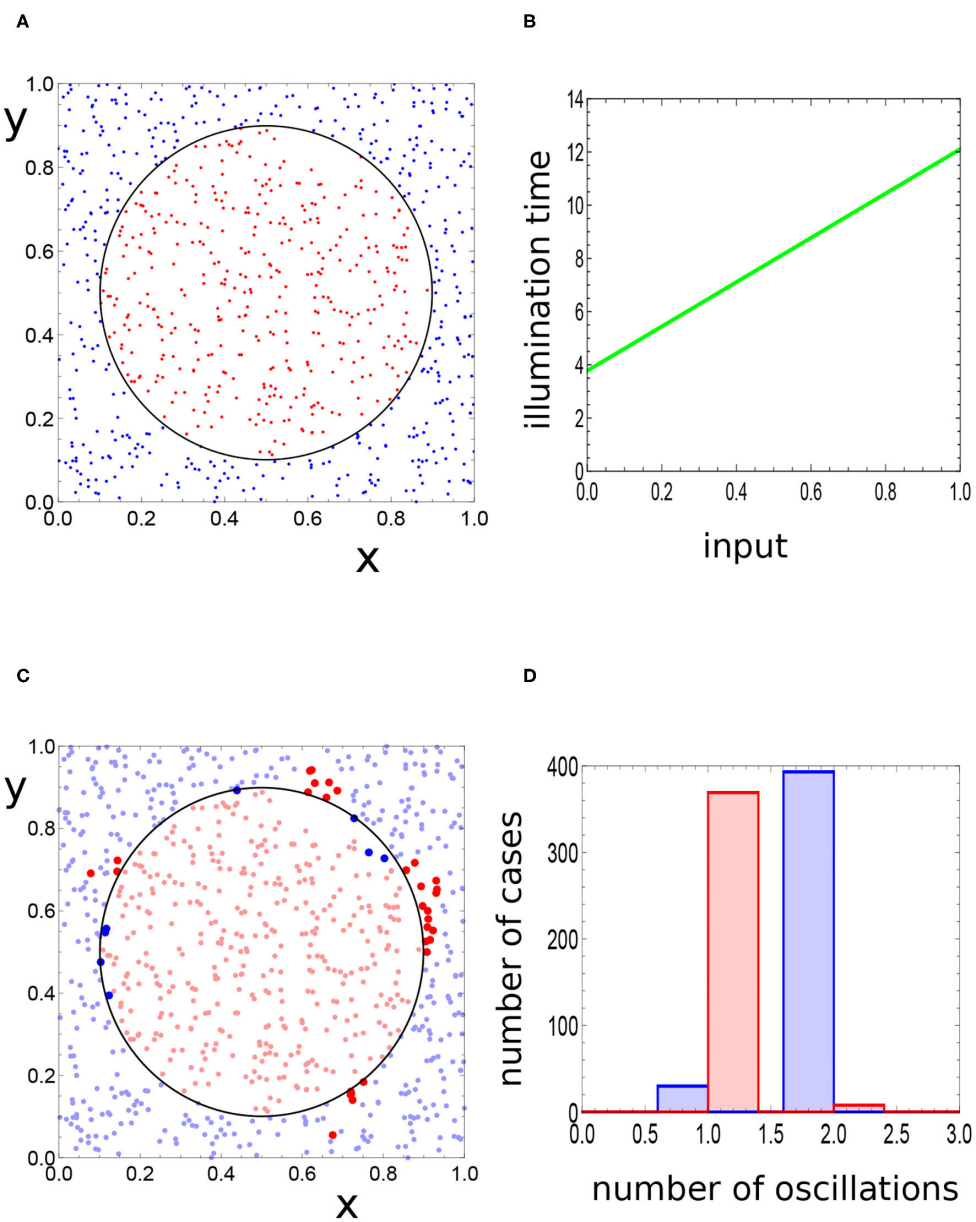
**(A)** Locations of 800 points representing the records of the training dataset *D*_*S*_. The red points are in the sun area, the blue ones outside it. **(B)** The relationship between *t*_*ilum*_ and the input value, obtained for the optimized network solving the Japanese flag problem. **(C)** The response of the optimized network to inputs from the training dataset. The dark red points are located outside the sun area and generate a single maximum of activator at the output oscillator. The dark blue points are in the sun area and generate two maxima of activator. **(D)** The red and blue bars correspond to the red and blue points in **(A)**. The majority of red points produce a single maximum of the activator on oscillator #3, whereas most of the records corresponding to blue points generate two maxima.

The method of the top-down optimization of a computing network has been described in details in our previous papers (Gizynski and Gorecki, 2016, 2017b), and we thus present it here shortly. In order to apply the method, we need a training dataset *D*_*S*_ = {(*x*_*n*_, *y*_*n*_, *g*_*n*_), *n* = 1, *N*} of the records in the form (*x, y, g*) where *x, y* ∈ [0, 1] and *g* ∈ {0, 1}. The numbers *x, y* denote the point coordinates and *g* is the record type; *g* = 1 for points located in the red disk and *g* = 0 for points outside it. Here, we used *D*_*S*_ of *N* = 800 records with randomly generated points inside [0, 1] × [0, 1] located as illustrated in Figure 3A. Let us also introduce the discrete random variable of record types *G*, defined as: *G* = {*g*_*n*_, *n* = 1, *N*}.

We postulate that information about the point color can be extracted from the number of activator maxima recorded on a selected oscillator of the network during the time interval [0, *t*_*max*_]. The quality of an oscillator network for solving a specific problem can be estimated in the following way. Let us consider a record (*x*_*n*_, *y*_*n*_, *g*_*n*_) ∈ *D*_*S*_ and study the network evolution for the input (*x*_*n*_, *y*_*n*_). Assume that *o*_1_(*n*), *o*_2_(*n*) and *o*_3_(*n*) are the numbers of activator maxima observed on oscillators #1, #2, and #3, respectively. Now, let us combine the results obtained for all inputs from the training dataset together and introduce the random variables *O*_*j*_ = {*o*_*j*_(*n*), *n* = 1, *N*} for *j* ∈ {1, 2, 3}. The mutual information between the random variables *G* and *O*_*j*_ is defined as (Cover and Thomas, 2006)

(11) $$\begin{array}{l} I\left(G;O_{j}\right)=H\left(G\right)+H\left(O_{j}\right)-H\left(G,O_{j}\right), \end{array}$$

and it measures the usefulness of the oscillator #j to give information on the problem output. In general, the mutual information is the amount of information (in bits) that one random variable contains about another random variable. In Equation (11), *H*(*A*) is the Shannon information entropy of the discrete random variable *A* (Shannon, 1948) and the random variable *G, O*_*j*_ is defined as *G, O*_*j*_ = {(*g*_*n*_, *o*_*j*_(*n*)), *n* = 1, *N*}. Obviously, the oscillator for which *I*(*G*; *O*_*j*_) is maximal was selected as the network output. The maximum *I*(*G*; *O*_*j*_) was used as the measure of network fitness in our optimization program.

The use of mutual information gives the quantitative measure of the network usefulness without the need to specify how to translate the number of activator maxima into the output. On the other hand, it has been shown that there is no monotonic relationship between the accuracy and mutual information (Gorecki, 2020), and a classifier optimized for the highest mutual information may therefore be less accurate than another one with lower mutual information. However, we believe this does not lead to a significant reduction in accuracy.

The network parameters, such as the type of oscillator #3, its inhibition time, the method for inputting the predictor values, or the type of interactions between oscillators, undergo optimization to achieve the highest mutual information on a representative dataset of cases. Both systematic methods of optimization and random trial and error ones can be applied. We have found (Gizynski and Gorecki, 2016, 2017b; Gizynski et al., 2017) that evolutionary optimization (Goldberg, 1989) oriented on obtaining the best classifier for a representative training dataset of the problem can lead to a computing network that performs the anticipated task with reasonable accuracy. At the beginning of the optimization procedure, we generated a population of *K* = 200 networks and selected a random training dataset illustrated in Figure 3A. The training dataset contained 377 points in the sun area (red) and 423 points in the surrounding region (blue). The adjustable parameters defining each network [type of oscillator #3, *t*_*ilum*_(3), *t*_*max*_, *t*_*start*_, *t*_*end*_, α and β] were randomly generated. The fitness of each network was calculated using the whole training dataset. The next generation comprised of 20% of most fit networks of the previous population and of 80% of offspring generated by recombination and mutation operations applied to oscillators from top 50% networks of the previous population. We randomly selected two parents from 50% of the fittest networks and next recombined randomly their parameters to obtain an offspring. After recombination, we applied mutations to randomly selected parameters. The probability of this operation was selected, such that on average, a single parameter of the network was mutated. The maximum change in the chosen parameter value was restricted to 10% of the original one. Next, the fit of networks belonging to the new generation were calculated, and the procedure was repeated. The optimization procedure was continued for 1,000 generations.

## 3. Results

The optimization procedure returned the network illustrated in Figure 2A in which the oscillator #3 is of the normal type and *t*_*ilum*_(3) = 6.37. The other parameters of the network are the following: *t*_*max*_ = 20.23, *t*_*start*_ = 3.78, *t*_*end*_ = 12.10, α = 0.849, and β = 0.251. The oscillator #3 is also the output one. For each input, we observed one or two activator maxima at the output oscillator. Figure 3C illustrates the relationship between the point location and the number of activator maxima. The statistic of the network outputs for all inputs from the training dataset is illustrated in Figure 3D. For most cases located in the sun area, we observed a single maximum (369 cases) and two maxima for only eight points (marked by dark blue dots in Figure 3C). On the other hand, for most of the background points, two activator maxima were observed (393 cases), whereas only 30 cases produced a single maximum (dark red points in Figure 3C). We can use the majority rule and declare that all points for which the network outputs a single maximum correspond to the sun, and all points for which two maxima are observed correspond to the surrounding white area. For the training dataset *D*_*S*_, such a majority rule leads to (369 + 393)/800 ~ 0.95 accuracy. It is interesting that the distribution of incorrectly attributed cases is not rotationally symmetric.

**Figure 4:**
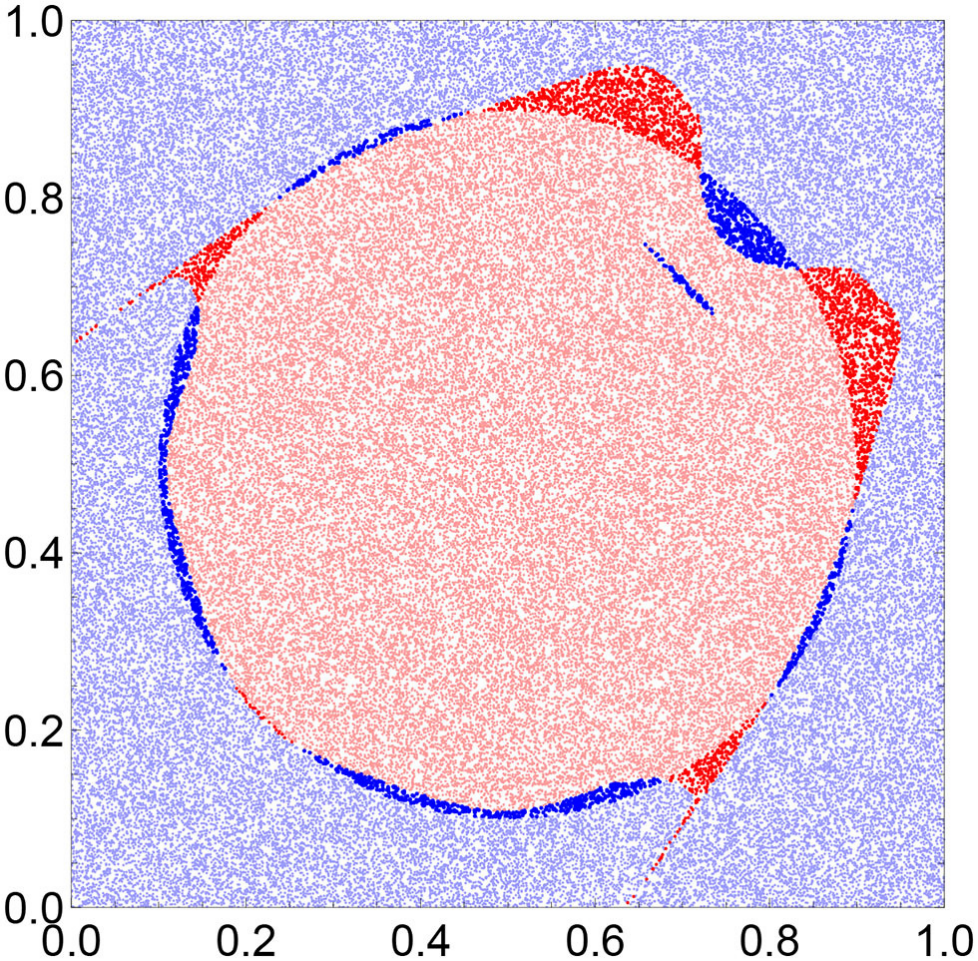
The figure shows how the optimized network sees the Japanese flag. Each dot corresponds to a record from the testing dataset that contains 100,000 records. The red points produce a single activator maximum and are thus considered to be sun. For the records represented by the blue points, two maxima are observed, and they are thus classified as the surrounding white area. The light red and light blue points are those that are classified correctly. The dark red points belong to the white region in Figure 1A, but the network thinks they belong to the sun. The dark blue points are the points belonging to the sun, but the network incorrectly classifies them as the points of the white surrounding area.

For more objective evaluation of the accuracy of the optimized network we considered a large test dataset *D*_*T*_ of 100,000 random, uniformly distributed points in the square [0, 1] × [0, 1]. Figure 4 shows the comparison between the location of a point from *D*_*T*_ and the number of activator maxima observed on the output oscillator within the time interval [0, *t*_*max*_]. The red light points are located inside the sun disk and produced a single maximum (48,028 cases). The light blue points are located in the surrounding area, and they forced two maxima of the output oscillator (47,117 cases). Using the majority rule introduced for the training dataset *D*_*S*_ we can say that the total number of correctly located points was 95,145 thus the classifier accuracy is ~95%. The dark colors mark points that are incorrectly attributed. The dark red points are located outside the sun, but they force a single activator maximum (2,967 cases). The dark blue points produced two activator maxima, but they were located in the sun area (1,888 cases).

**Figure 5:**
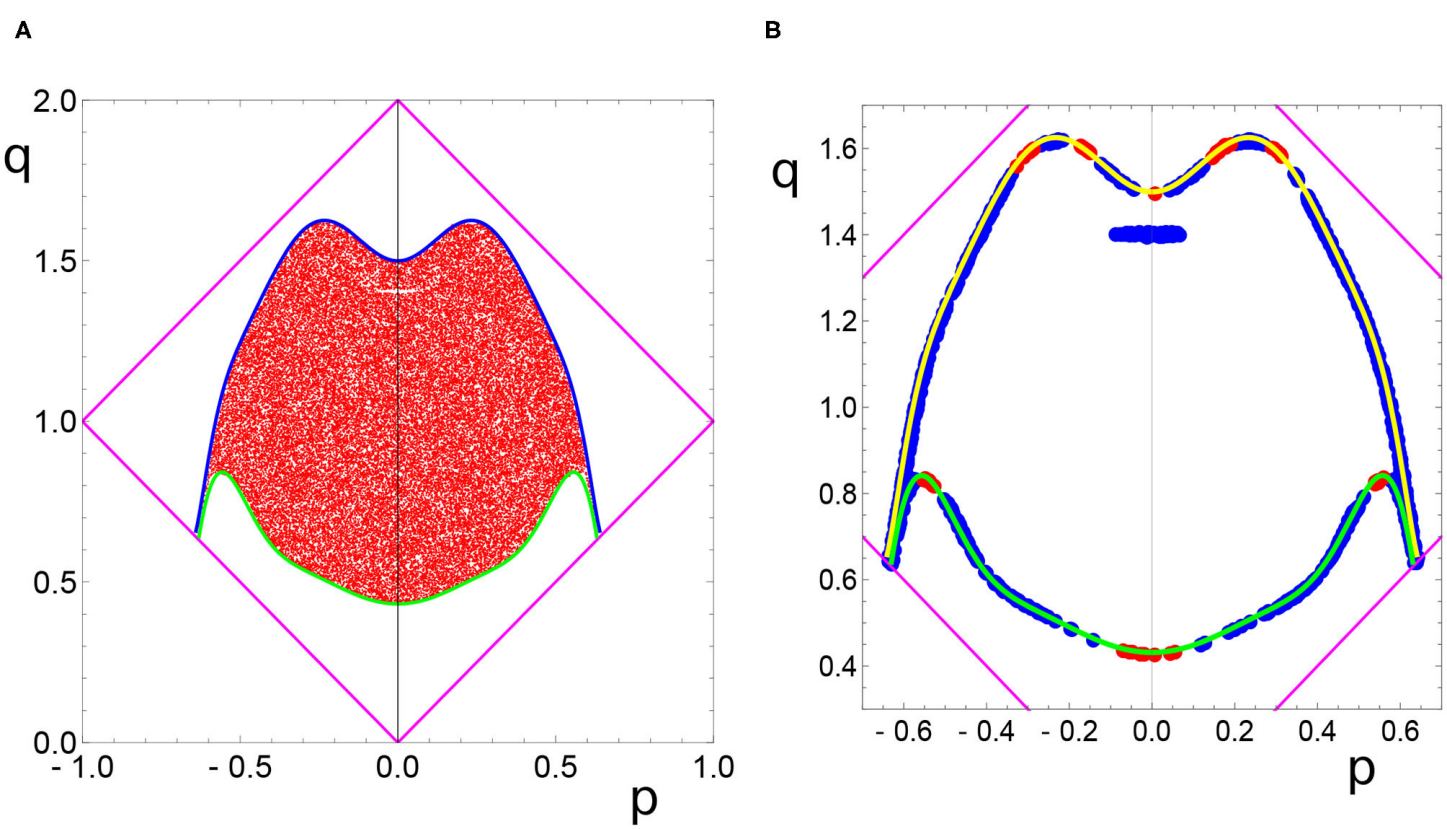
**(A)** The shape of the sun area, as seen by the optimized network in the *p, q* coordinates. The blue and green lines are the fits of the sun boundaries (cf. Equations 12, 13). **(B)** The positions of incorrectly classified points that are located in the region between the fitting lines. The blue points are classified as belonging to the region outside, but they should be inside. The red points are classified as belonging to the red region, but they fail outside the fitting lines. In **(B)** for better visibility, the upper fitting line is represented by a yellow curve.

The red points in Figure 4 (both light and dark) can be regarded as the image of the sun area seen by the optimized network. Instead of the disk, the network senses a complex, two-horned shape. In order to describe it more precisely we introduce new coordinates: *p* = *x* − *y* and *q* = *x* + *y*. In these coordinates, the points producing a single activator maximum on the output oscillator are located, as shown in Figure 5A. The upper fitting curve (blue) is described:

(12) $$\begin{array}{l} F_{U}\left(p\right)=1.49928+5.56776p^{2}-81.5484p^{4}+503.745p^{6}\\ \;-2275.64p^{8}+7512.65p^{10}-14690.6p^{12}+11764.8p^{14} \end{array}$$

and the lower fitting curve (green) is:

(13) $$\begin{array}{l} F_{D}\left(p\right)=0.432601+1.65454p^{2}-2.0677p^{4}-107.24p^{6}\\ \;+1120.59p^{8}-3887.3p^{10}+5377.69p^{12}-2552.69p^{14} \end{array}$$

Now we can forget about the original problem and consider the question if a chemical computer can correctly distinguish the points of the unit square that are located between the curves *F*_*D*_(*x* − *y*) and *F*_*U*_(*x* − *y*). Such a problem looks rather difficult, but the answer is simple: a good candidate for such a chemical computer is the optimized network described above. Its accuracy is around 99%. The incorrectly attributed points are marked in Figure 5B. Most of them are located at the boundary, and the error may be connected with oversimplified fitting. There are also some points for which the attribution error is hard to explain (the blue horizontal line at *q* ~ 1.4 on Figure 5B), It would be interesting to verify if the number of such points can be reduced by changing the parameters of Oregonator.

## 4. Discussion and Conclusions

In this paper, we investigated whether a chemical computer can solve the color determination problem for a point on the Japanese flag. We considered the flag of Japan because it represents more complex geometrical structure than the striped pattern common for many flags like the Polish or the French ones. For example the flag of France can be represented by a unit square with the blue points for (*x, y*), *x* ∈ [0, 1/3), the white area for (*x, y*), *x* ∈ [1/3, 2/3), and the red region defined as (*x, y*), *x* ∈ [2/3, 1]. In such a case, the point color can be easily determined by a single oscillator that is also the input of x-coordinate. Let us consider *t*_*start*_ = 0. The inhibition time of input oscillator [*t*_*illum*_(1)] is proportional to the value of *x* [*t*_*illum*_(1) = *xt*_*end*_], and we can thus select *t*_*end*_ such that a small number of oscillations appear in the red area, more of them are seen for the white points, and the largest number of oscillations is observed if the input represents coordinates of a blue point. For example, if *t*_*end*_ = 32.4, then the oscillator with parameters given in this paper observed for *t*_*max*_ = 47 shows 4, 3, and 2 maxima of activator for blue, white and red regions respectively.

The idea of a neural network has inspired the considered medium structure, composed as it is of interacting, individual units. Classical image recognition methods are based on multilayered neural networks in which the output of an artificial neuron is a single number (MacKay, 2003). In our approach, the time evolution of a neuron (oscillator) is more complex. The output of a separated artificial neuron remains constant, whereas, in our medium, the activator concentration in a non-interacting oscillator periodically changes in time. The output also depends on the time *t*_*max*_ it was observed. It seems that to determine the color of a point on the Japanese flag with a given accuracy with a standard neural network, one needs more nodes than the number of oscillators used by our medium (Zammataro, 2010).

We have demonstrated that a simple network of just three coupled chemical oscillators can predict the color of a randomly selected point on the Japanese flag with 95% accuracy. Another interesting result is that the network delivers a fast answer. The output information if a point belongs to the white or the red region on the flag appears just within two oscillation periods. Our simulations were based on the Oregonator model that is more realistic than the event-based model previously used in papers on chemical classifiers (Gizynski and Gorecki, 2016, 2017b; Gizynski et al., 2017; Gorecki, 2020). The results of both models were qualitatively similar. It confirms that even small networks of interacting oscillators can perform complex computations. It can be expected that a more accurate location of point color can be achieved when more oscillators are taken into account. However, the numerical complexity of optimization rapidly increases with the number of parameters. We are working on the optimization method for large networks that would include previously accumulated results for smaller ones.

It is worth noticing that the points classified as belonging to the sun area group into an interesting, horned shape illustrated in Figure 4A. The boundaries of this shape can be described by complex polynomials of a high order. The problem of finding whether a point is located inside a horn-shaped area is more complex than the determination of point location with respect to a disk located at the center of the unit square. If a point belongs to the horned region, a high accuracy algorithm (~99% accuracy) is given by the network of three oscillators we optimized to see the Japanese flag. We doubt whether any multilayered neural network can produce equally simple algorithm of finding a point in the horn-shaped area. An interesting problem for future studies is whether or not the shape of the correctly classified region can be regulated by the network parameters.

Results presented in the paper were obtained based on computer simulations, but the Oregonator model can qualitatively describe BZ-reaction and therefore brings information for potential experiments on the chemical computation. Systems of interacting oscillators have been studied experimentally using a few techniques (Vanag and Epstein, 2004; Kaminaga et al., 2005, 2006; Gentili et al., 2017). Interacting droplets containing reagents of BZ-reaction can be stabilized by a solution of lipids in the organic phase (Szymanski et al., 2011). If the photosensitive variant of BZ-reaction is used, then oscillations in droplets can be individually controlled. Experiments on the control of three coupled droplets mechanically stabilized inside a plastic cage were reported in Gizynski and Gorecki (2017a). Precisely the same system can be used as the classifier of a point in the Japan flag. The droplets acting as normal oscillators in the network are inhibited by illumination within the time interval that does not depend on the input values. The illumination times of input droplets depend on *x* and *y*, as described by Equations (9) and (10). However, the experiments have demonstrated (Gizynski and Gorecki, 2017a) that it is rather difficult to stabilize even three droplets if a standard variant of BZ-reaction with the malonic acid is used. The bubbles of gas can appear between droplets, deform them, and change interactions between oscillators. We therefore believe that solid objects loaded with the catalyst, like DOWEX beads (Kuze et al., 2019) or silica gel beads (Mallphanov and Vanag, 2020), seem to be more suitable for experiments with information processing using a network of oscillators. Also, the strategy of optical communication described by Gentili et al. (2017) can be applied to control interactions between oscillators and the inflow of input data.

We believe the maximization of information processing functionality based on optimization of the mutual information can be combined with other types of complex chemical dynamics, such as excitability or multistability. However, in typical experiments, oscillations are robust, whereas other types of non-linear behavior are more difficult to control, stabilize, and repeat. Additionally, our results illustrate that even a small network of oscillators can have significant information processing potential.

## Data Availability Statement

All datasets presented in this study are included in the article.

## Author Contributions

JG was responsible for the idea of the presented study, network optimization, and presentation of results. AB helped to develop the model for interactions between oscillators and verified the obtained results using her own simulation code. All authors contributed to the article and approved the submitted version.

## Conflict of Interest

The authors declare that the research was conducted in the absence of any commercial or financial relationships that could be construed as a potential conflict of interest.

## References

[B1] Adamatzky A. (ed.). (2018). Advances in Uncnoventional Computing. Vol. 2 Springer.

[B2] Adamatzky A. De Lacy Costello B. (2002). Experimental logical gates in a reaction diffusion medium: the XOR gate and beyond. Phys. Rev. E 66:046112 10.1103/PhysRevE.66.046112 12443264

[B3] Adamatzky A. De Lacy Costello B. Asai T. (2005). Reaction-Diffusion Computers. New York, NY: Elsevier.

[B4] Adamatzky A. De Lacy Costello B. Bull L. Holley J. (2011). Towards arithmetic circuits in subexcitable chemical media. Isr. J. Chem. 51, 56–66. 10.1002/ijch.201000046

[B5] Adleman L. M. (1994). Molecular computation of solutions to Combinatorial Problems. Science 266, 1021–1024. 7973651 10.1126/science.7973651

[B6] Agladze K. Magome N. Aliev R. Yamaguchi T. Yoshikawa K. (1997). Finding the optimal path with the aid of chemical wave. Phys. D 106, 247–254. 10.1016/S0167-2789(97)00049-3

[B7] Belousov B. P. (1959). Periodically acting reaction and its mechanism (in Russian), in Collection of Short Papers on Radiation Medicine (Medgiz), 145–152.

[B8] Calude C. S. (2002). Computing with Cells and Atoms. London: Taylor & Francis Publishers.

[B9] Cover T. M. Thomas J. A. (2006). Elements of Information Theory. New York, NY: Wiley-Interscience.

[B10] de Silva A. P. Uchiyama S. (2007). Molecular logic and computing. Nat. Nanotechnol. 2, 399–410. 10.1038/nnano.2007.188 18654323

[B11] Epstein I. R. Pojman J. A. (1998). An Introduction to Nonlinear Chemical Dynamics: Oscillations, Waves, Patterns, and Chaos. New York, NY: Oxford University Press.

[B12] Feynman R. P. Hey T. Allen R. (2000). Feynman Lectures on Computation. Boulder, CO: CRC Press.

[B13] Field R. J. Burger M. (eds.). (1985). Oscillations and Traveling Waves in Chemical Systems. New York, NY: Wiley. 10.1126/science.229.4716.852 17777924

[B14] Field R. J. Noyes R. M. (1974). Oscillations in chemical systems. IV. Limit cycle behavior in a model of a real chemical reaction. J. Chem. Phys. 60, 1877–1884.

[B15] Gentili P. L. Giubila M. S. Germani R. Romani A. Nicoziani A. Spalletti A.. (2017). Optical communication among oscillatory reactions and photo-excitable systems: UV and visible radiation can synchronize artificial neuron models. Angew. Chem. Int. Ed. 56, 7535–7540. 10.1002/anie.201702289 28560808

[B16] Gizynski K. Gorecki J. (2016). A chemical system that recognizes the shape of a sphere. Comput. Methods Sci. and Technol. 22, 167–177. 10.12921/cmst.2016.0000057

[B17] Gizynski K. Gorecki J. (2017a). Chemical memory with states coded in light controlled oscillations of interacting Belousov-Zhabotinsky droplets. Phys. Chem. Chem. Phys. 19, 6519–6531. 10.1039/C6CP07492H 28197558

[B18] Gizynski K. Gorecki K. (2017b). Cancer classification with a network of chemical oscillators. Phys. Chem. Chem. Phys. 19, 28808–28819. 10.1039/C7CP05655A 29051945

[B19] Gizynski K. Gruenert G. Dittrich P. Gorecki J. (2017). Evolutionary design of classifiers made of oscillators containing a nonlinear chemical medium. MIT Evol. Comput. 25, 643–671. 10.1162/EVCO_a_00197 27728772

[B20] Goldberg D. E. (1989). Genetic Algorithms in Search, Optimization and Machine Learning. Boston, MA: Addison-Wesley Longman Publishing Co., Inc.

[B21] Gorecka J. Gorecki J. (2006). Multi-argument logical operations performed with excitable chemical medium. J. Chem. Phys. 124:084101. 10.1063/1.2170076 16512702

[B22] Gorecki J. (2020). Applications of information theory methods for evolutionary optimization of chemical computers. Entropy 22:313 10.3390/e22030313 PMC7516772 33286087

[B23] Gorecki J. Gorecka J. N. Igarashi Y. (2009). Information processing with structured excitable medium. Nat. Comput. 8, 473–492. 10.1007/s11047-009-9119-y24827316

[B24] Grüunert G. Gizynski K. Escuela G. Ibrahim B. Gorecki J. Dittrich P. (2015). Understanding networks of computing chemical droplet neurons based on information flow. Int. J. Neur. Syst. 25:1450032. 10.1142/S0129065714500324 25476910

[B25] Hjelmfelt A. Weinberger E. D. Ross J. (1992). Chemical implementation of finite-state machines. Proc. Natl. Acad. Sci. U.S.A. 89, 383–387. 11607249 10.1073/pnas.89.1.383 PMC48241

[B26] Holley J. Jahan I. De Lacy Costello B. Bull L. Adamatzky A. (2011). Logical and arithmetic circuits in Belousov-Zhabotinsky encapsulated disks. Phys. Rev. E 84:056110. 10.1103/PhysRevE.84.056110 22181476

[B27] Jung P. Cornell-Bell A. Moss F. Kadar S. Wang J. Showalter K. (1998). Noise sustained waves in subexcitable media: from chemical waves to brain waves. Chaos 8, 567–575. 10.1063/1.166338 12779760

[B28] Kádár S. Amemiya T. Showalter K. (1997). Reaction Mechanism for Light Sensitivity of the ${\text{Ru}(\text{bpy})}_{3}^{2+}$ -Catalyzed Belousov-Zhabotinsky Reaction. J. Phys. Chem. A 101, 8200–8206.

[B29] Kaminaga A. Vanag V.K. Epstein I. R. (2006). A reaction-diffusion memory device. Angew. Chem. Int. Ed. 45, 3087–3089. 10.1002/anie.200600400 16570336

[B30] Kaminaga A. Vanag V. K. Epstein I. R. (2005). “Black spots” in a surfactant-rich Belousov-Zhabotinsky reaction dispersed in a water-in-oil microemulsion system. J. Chem. Phys. 122:174706. 10.1063/1.1888386 15910059

[B31] Kuhnert L. (1986). A new optical photochemical memory device in a light-sensitive chemical active medium. Nature 319, 393–394. 10.1038/319393a0

[B32] Kuhnert L. Agladze K. I. Krinsky V. I. (1989). Image processing using light-sensitive chemical waves. Nature 337, 244–247. 10.1038/337244a0

[B33] Kuze M. Horisaka M. Suematsu N. J. Amemiya T. Steinbock O. Nakata S. (2019). Chemical wave propagation in the Belousov-Zhabotinsky reaction controlled by electrical potential. J. Phys. Chem. A 123, 4853–4857. 10.1021/acs.jpca.9b02636 31094190

[B34] Lin X. Liu Y. Deng J. Lyu Y. Qian P. Lia Y.. (2018). Multiple advanced logic gates made of DNA-Ag nanocluster and the application for intelligent detection of pathogenic bacterial genes. Chem. Sci. 9, 1774–1781. 10.1039/C7SC05246D 29675221 PMC5892130

[B35] MacKay D. J. C. (2003). Information Theory, Inference and Learning Algorithms. Cambridge, UK: Cambridge University Press.

[B36] Magri D. C. Brown G. J. McClean G. D. de Silva A. P. (2006). Communicating chemical congregation: a molecular AND logic gate with three chemical inputs as a “lab-on-a-molecule” prototype. J. Am. Chem. Soc. 128, 4950–4951. 10.1021/ja058295+ 16608318

[B37] Mallphanov I. L. Vanag V. K. (2020). Fabrication of new Belousov-Zhabotinsky micro-oscillators on the basis of silica gel beads. J. Phys. Chem. A 124, 272–282. 10.1021/acs.jpca.9b09127 31899640

[B38] McKinney B. O. F. Daly B. Yao C. Schroeder M. de Silva A. P. (2017). Consolidating molecular logic with new solid-bound YES and PASS 1 gates and their combinations. ChemPhysChem 18:1760. 10.1002/cphc.201700120 28349591

[B39] Press W. H. Teukolsky S. A. Vetterling W. T. Flannery B. P. (2007). Numerical Recipes: The Art of Scientific Computing. New York, NY: Cambridge University Press.

[B40] Rambidi N. G. Maximychev A. V. (1997). Towards a biomolecular computer. Information processing capabilities of biomolecular nonlinear dynamic media. Biosystems 41, 195–211. 10.1016/S0303-2647(96)01678-4 9113354

[B41] Shannon C. E. (1948). A mathematical theory of communication. Bell Syst. Tech. J. 27, 379–423. 10.1002/j.1538-7305.1948.tb01338.x

[B42] Smelov P. S. Vanag V. K. (2017). Experimental investigation of a unidirectional network of four chemical oscillators pulse-coupled through an inhibitor. Russ. J. Phys. Chem. A 91, 1015–1020. 10.1134/S003602441706022X

[B43] Steinbock O. Kettunen P. Showalter K. (1996). Chemical wave logic gates. J. Phys. Chem. 100, 18970–18975. 10.1021/jp961209v

[B44] Steinbock O. Toth A. Showalter K. (1995). Navigating complex labyrinths-optimal paths from chemical waves. Science 267, 868–871. 10.1126/science.267.5199.868 17813917

[B45] Szymanski J. Gorecka J. N. Igarashi Y. Gizynski K. Gorecki J. Zauner K. P. (2011). Droplets with information processing ability. Int. J. Unconv. Comput. 7, 185–200.

[B46] Toth A. Showalter K. (1995). Logic gates in excitable media. J. Chem. Phys. 103, 2058–2066. 10.1063/1.469732

[B47] Vanag V. K. Epstein I. R. (2004). Stationary and oscillatory localized patterns, and subcritical bifurcations. Phys. Rev. Lett. 92:128301. 10.1103/physrevlett.92.128301 15089714

[B48] Vanag V. K. Yasuk V. O. (2018). Dynamic modes in a network of five oscillators with inhibitory all-to-all pulse coupling. Chaos 28:033105. 10.1063/1.5004015 29604639

[B49] Zammataro L. (2010). Solving the Hole in the Square Problem with a Neural Network. Available online at: http://demonstrations.wolfram.com/SolvingTheHoleInTheSquareProblemWithANeuralNetwork/

[B50] Zhabotinsky A. M. (1964). Periodic oxidizing reactions in the liquid phase. Dokl. Akad. Nauk SSSR 157, 392–395.

